# Risk Factors and Incidence Rates of Self-Reported Short-Term Adverse Events of COVID-19 Vaccine Booster Dose

**DOI:** 10.3390/vaccines10071115

**Published:** 2022-07-13

**Authors:** Po-Yu Chen, Bih-Ju Wu, Mei-Chin Su, Yen-Hsi Lin, Shu-Chiung Chiang, Jau-Ching Wu, Tzeng-Ji Chen, Yu-Chun Chen

**Affiliations:** 1 Department of Family Medicine, Taipei Veterans General Hospital, Taipei 112, Taiwan; barry50710@gmail.com (P.-Y.C.); sonia1999082727@yahoo.com (Y.-H.L.); tjchen@vhct.gov.tw (T.-J.C.); 2 School of Medicine, National Yang Ming Chiao Tung University, Taipei 112, Taiwan; jauching@gmail.com; 3 Department of Nursing, Taipei Veterans General Hospital, Taipei 112, Taiwan; bjwu@vghtpe.gov.tw (B.-J.W.); mcsu@vghtpe.gov.tw (M.-C.S.); 4 Institute of Hospital and Health Care Administration, School of Medicine, National Yang Ming Chiao Tung University, Taipei 112, Taiwan; scchiang0g@gmail.com; 5 Department of Neurosurgery, Neurological Institute, Taipei Veterans General Hospital, Taipei 112, Taiwan; 6 Big Data Center, Taipei Veterans General Hospital, Taipei 112, Taiwan; 7 Department of Family Medicine, Taipei Veterans General Hospital, Hsinchu Branch, Hsinchu 31064, Taiwan

**Keywords:** COVID-19 vaccines, adverse events (AE)/reaction, short-term serious adverse events (SAE), short-term non-serious adverse events (NSAE), vaccine adverse event reporting system (VAERS), booster vaccination, mix-and-match

## Abstract

With the spread of the new SARS-CoV-2 variants, many countries have begun COVID-19 vaccine booster programs with the mix-and-match strategy. However, research on the adverse events (AE) of booster doses is still scarce. The aim of our study was to analyze the reported incidence rate (IR), and factors associated with AE, including short-term serious adverse events (SAE) and short-term non-serious adverse events (NSAE), among different vaccine products through the hospital-based Vaccine Adverse Event Reporting System (VAERS). A total of 7432 records were collected during the three-month study period. While more than half of the responses (52.2%) reported the presence of AE after receiving a booster dose, only a few AE were considered SAE (2.4%). AE were significantly higher among women and people of younger age, and the brand of vaccines is the strongest factor associated with post-booster dose AE. The incidence of AE in mRNA1273 is higher than in BNT162b2 and MVC-COV1901 (IRR mRNA1273 vs. BNT162b2: 1.22, 95% CI: 1.11–1.34; BNT162b2 vs. MVC-COV1901: 2.77, 95% CI: 2.27–3.39). The IR of different groups were calculated to support the decision making of the booster vaccine. Although AE were not uncommon for booster vaccines, almost all AE were not serious and predictable using estimated IR. This result can be used to optimize booster vaccine decision making.

## 1. Introduction

The concern about the safety and side effects of the COVID-19 vaccines is considered to be the main impediment to the effectiveness of the public COIVD-19 vaccination program [1,2]. Side effects, even adverse events (AE) of product-class effects and mix-and-match were often reported and inquired about. The lack of post-vaccination surveillance and real-world data support made it difficult to select the most appropriate vaccine [3]. Although many countries had national or hospital-based self-reporting surveillance systems in place, such as the Vaccine Adverse Event Reporting System (VAERS) and Vaccine Safety Datalink (VAS) in the United States and the Yellow Card Scheme in the United Kingdom [4,5], only a few studies examined the AE of the mix-and-match booster method [6,7]. As more and more brands of the COVID-19 vaccine were introduced into clinical use, multiple combinations of primary boosters were presented. The study about the comparison of AE among different combinations was crucial in determining the best option.

Taiwan had a lower confirmed COVID-19 rate of 32,217 per million population (compared to the global rate of 66,175 per million population), and a higher vaccination coverage rate (first and second doses: 85.86% and 80.76%, respectively, compared to the global rate of 59.81% with primary series). The vaccination coverage rate for booster vaccine has reached 62.77%, since the booster dose vaccination program was initiated on 2 December 2021 [8,9]. Because of the abundant supply of vaccines, the booster dose program allowed Taiwanese people to select the most suitable brand of COVID-19 vaccines as the booster dose from the following four brands: AstraZeneca ChAdOx1 (AZD1222), Moderna mRNA1273 (Spikevax), Pfizer-BioNTech BNT162b2 and MVC (Medigen Vaccine Biologics Corporation) MVC-COV1901. The first three vaccines are on the WHO Emergency Use List (EUL) and the last is a protein subunit COVID-19 vaccine developed by Medigen Vaccine Biologics Corporation in Taiwan, an American company, Dynavax Technologies and the U.S. National Institutes of Health [10]. In addition to the national surveillance system VAERS, Taiwan’s hospitals were requested to set up hospital-based AE tracking systems [11].

Realizing that post-vaccination data can reduce skepticism and promote vaccination policy [12,13], our research aimed to analyze the reported incidence rate (IR) for AE, including short-term serious adverse events (SAE) and short-term non-serious adverse events (NSAE) of booster vaccines, and factors associated with AE between different vaccine products through self-reported hospital VAERS. We conducted the study with data from Taipei Veterans General Hospital (TVGH), one of the largest hospitals in Taiwan. The result of this research can be a significant reference for general people to select the most suitable brand of booster dose for themselves.

## 2. Materials and Methods 

### 2.1. Overview

#### 2.1.1. Booster Vaccination Program in Taiwan

Taking into account the effect of immunization, Taiwan’s Central Epidemic Command Center (CECC) announced the start of the booster vaccination program on 2 December 2021. People can freely select a booster dose among four different brands, including viral particles of ChAdOx1 2.5 × 10^8^/0.5 mL/dose, mRNA1273 50 μg/0.25 mL/dose, BNT162b2 30 μg/0.3 mL/dose and MVC-COV1901 15 μg/0.5 mL/dose (Appendix A Table A1). The CECC suggested that if people received viral vector vaccines as the primary vaccination schemes, such as ChAdOx1 and Janssen COVID-19 vaccine, they should select mRNA1273 or BNT162b2 as a booster dose. It is noticed that the booster dose amount of mRNA1273 was 50 μg, only half of the primary series (100 μg). The other booster dose amounts of the other three brands were the same as those of the primary series in Taiwan [8].

#### 2.1.2. VAERS in Taipei Veterans General Hospital (TVGH)

TVGH set up a vaccination site. It supplies over 10,000 booster doses of vaccinations per month. As a result, we created an online anonymous questionnaire for the TVGH’s hospital-based VAERS. Google Forms was used to produce the questionnaire, which was written in traditional Chinese. We printed it out as a QR code on the paper note, which also included a reminder to visit a clinic or hospital if the recipients experienced significant discomfort after the vaccination. Doctors provided recipients with paper notes after they obtained doctor’s evaluations, asking them to report whether they experienced AE or not after 7 days of vaccination. The questionnaire was divided into two sections. The first section dealt with respondents’ personal information, such as gender and age. Respondents who answered “yes” to that discomfort were asked to move on to the next section. The second part was to collect information on the brand of COVID-19 vaccines they received, the self-reported AE experienced during the primary series and booster doses, as well as the method of handling them. We listed “fever”, “fatigue”, “pain/swelling at the injection site”, “headache”, “severe allergy” and “others” in the question “what symptoms do you have?” Respondents could choose multiple answers or explain their own symptoms. Using Google’s deduplicate process, each respondent was allowed to submit their survey only once.

### 2.2. Setting, Data Source, and Ethical Concerns

The study was carried out on vaccinees who had received the booster dose in TVGH. After 7 days of booster vaccination, they would be asked to report any adverse reactions. From 13 December 2021 to 13 March 2022, a total of 7431 replies were collected during the first three months of Taiwan’s booster program.

This study’s protocol was approved by the Institutional Review Board of Taipei Veterans General Hospital (IRB No. 202208001AE). The Institutional Review Board waived the necessity for written informed consent from each patient who participated in our study because the data we evaluated was de-identified. Furthermore, no personally identifiable information or human biospecimens were used.

### 2.3. Data Processing

**Figure 1:**
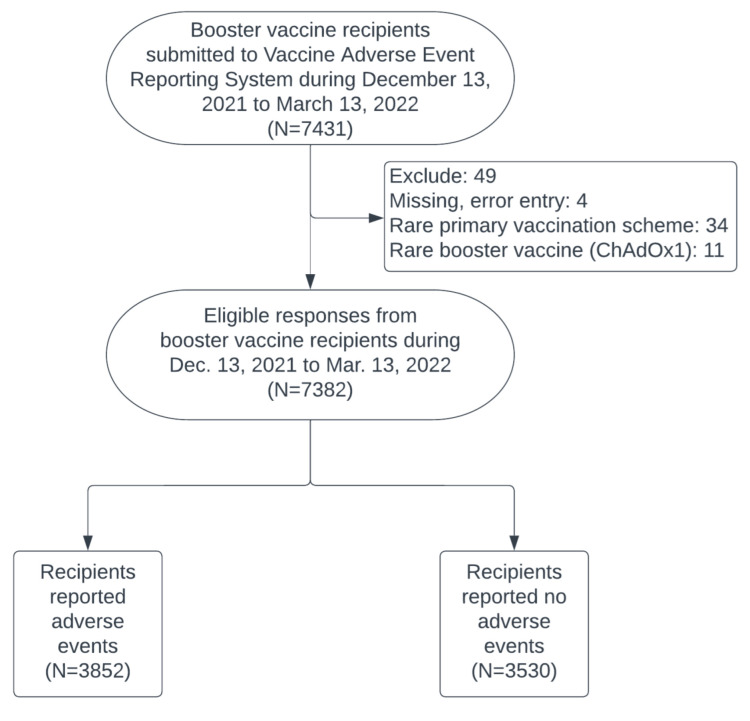
Processing flow of anonymous responses of booster vaccine recipients.

Anonymous responses submitted to VAERS of TVGH were collected from 13 December 2021 to 13 March 2022. We enrolled responses with the three most commonly used brands of booster vaccine, including mRNA1273, BNT162b2, and MVC-COV1901, since ChAdOx1 is rarely used. Furthermore, we excluded responses with missing data and responses reported with vaccination schemes with very few numbers in Taiwan. The detailed process is listed in Figure 1.

#### 2.3.1. Classification of AE, Short-Term Serious Adverse Events (SAE) and Short-Term Non-Serious Adverse Events (NSAE)

AE were identified according to the symptoms reported by the respondents with their own descriptions. The third author (M.-C.S.) would classify the multiple descriptions into eight categories: “local reactions”, “flu-like symptoms”, “cardiac symptoms”, “gastrointestinal symptoms”, “systemic allergic reactions”, “muscle/joint pain”, “menstrual problems” and “others” as previous research described [14]. Two authors (P.-Y.C. and Y.-C.C.) rechecked AE and grouped symptoms such as chest pain, short of breath and systematic allergic reactions as SAE, and left the rest symptoms as NSAE.

#### 2.3.2. Definition of Mix-and-Match Method

In Taiwan, COVID-19 vaccination with the mix-and-match method has been con-ducted since 11 August 2021. People can select any brand of vaccine as the primary series and booster dose [15,16]. In our study, the type of primary-booster vaccination was determined by serial use of homologous boosters (the same vaccine type as the last primary vaccine) and heterologous boosters (the different vaccine types from the last primary vaccine) in fully vaccinated recipients. For example, vaccinees can choose ChAdOx1 as the first dose, mRNA1273 as the second dose and MVC-COV1901 as the booster dose. The definitions and examples can be seen in Table 1.

### 2.4. Statistical Analysis

We calculated IR of AE as the sum of all reported adverse events divided by the number of respondents and expressed as the fraction of 100 respondents. Binominal 95% confidence intervals (CI) were calculated and used to compare IR of AE between different subgroups. A Poisson regression model was fitted and incidence rate ratios (IRR) were used to assess risk factors associated with AE. Furthermore, we used Poisson regression to estimate IR and 95% confidence intervals for every group of combination of risk factors. All the data were analyzed by Stata software (Stata Corp, College Station, TX, USA). A two-tailed level of 0.05 was considered statistically significant.

## 3. Results

### 3.1. Characteristics and Adverse Events (AE) Reported to Vaccine Adverse Event Reporting System (VAERS)

From 13 December 2021 to 13 March 2022, a total of 30,832 people received booster doses in the TVGH. Nearly a quarter of the responses (7382, 24.0%) were included in the hospital-based VAERS, and the characteristics are presented in Table 2. The percentage of female respondents (66.7%) was twice as it of male responders (33.3%). Respondents’ age range was primarily under 64 years old, according to the statistics (91.2%). Within the primary vaccination scheme, ChAdOx1/ChAdOx1 was the most common (59.7%), followed by mRNA1273/mRNA1273 (19.5%). Regarding the type of primary-booster combination, heterologous booster vaccination (71%) was prominently higher than homologous booster vaccination (29%). Regarding the brand of COVID-19 vaccination booster dose, more than two-thirds of the respondents (72.8%) received the mRNA1273 vaccine, while 16.0% of the respondents received the BNT162b2 vaccine, and 11.2% of the respondents received the MVC-COV1901.

Overall, over half of the responses (52.2%) reported the presence of AE after receiving a booster dose. Compared to the gender difference, females had a higher IR of AE (59%) than males (38.2%). We also found that respondents under 39 years of age had a higher IR of AE (61.9%) than those between 40 and 64 years of age (48.7%), and those over 65 years of age (26.3%). Among primary vaccination schemes, the IR of AE in ChAdOx1/mRNA1273 was the highest (64.5%), and MVC-COV1901/MVC-COV1901 was the lowest (26.9%). Between the types of primary-booster combinations, the IR of AE in heterologous booster vaccination (54.1%) was higher than in homologous booster vaccination (47.5%). The brand of booster dose was also an important factor for the IR of AE. The mRNA1273 was the major brand of booster dose, and its IR of AE (58.7%) was greater than BNT162b2 (47.2%) and MVC-COV1901 (16.2%).

**Figure 2:**
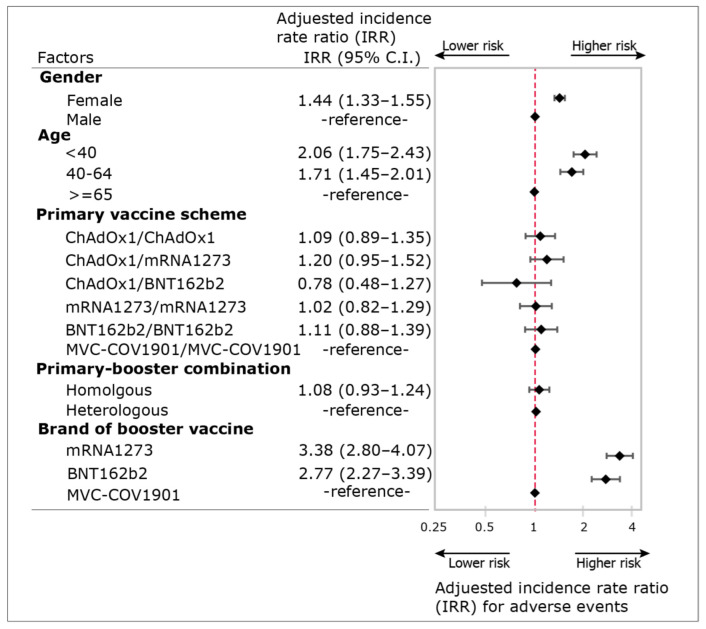
Adjusted incidence rate ratio (IRR), 95% confidence interval (95% C.I.) and forest plot of factors for adverse events (AE) reported to hospital-based vaccine adverse event reporting system (VAERS) for persons who respond to a booster dose of the COVID-19 vaccine from 13 December 2021 to 13 March 2022, at the vaccination station of the Taipei Veterans General Hospital, Taipei, Taiwan. (n = 7382, Taipei, Taiwan).

There were three risk factors: gender, age group and brand of booster dose, associated with the occurrence of AE after booster vaccines (Figure 2). Using the occurrence of any AE as the dependent variable and gender, age, primary vaccine scheme, primary-booster combination and brands of booster vaccine as the independent variables, the fitted Poisson model disclosed only gender, age group and brand of booster dose are all statistically significant factors associate with AE. The IRR in the brand of booster dose was evidently higher than sex and age, suggesting that the brand of booster dose was the single most important factor associated with AE. Furthermore, the incidence of AE of mRNA1273 and BNT162b2 was more than twice that of MVC-COV1901 after controlling other factors (IRR of mRNA1273: 3.38, IRR of BNT162b2: 2.77, both *p* < 0.001) (Figure 2).

### 3.2. Reports of Adverse Events (AE) to Vaccine Adverse Event Reporting System(VAERS) by COVID-19 Booster Vaccine Recipients

The incidence of AE considerably varied by COVID-19 booster vaccine brands (Table 3). While more than half (52.2%) of respondents reported post-booster vaccination AE, a small fraction (2.4%) were considered as serious. Both mRNA1273 and BNT162b2 vaccinees were at least two times more likely to have any AE than those of MVC-COV1901 (crude incidence rate ratio (cIRR); mRNA1273 vs. MVC-COV1901; 3.62 (95% C.I.: 3.05–4.30); BNT162b2 vs. MVC-COV1901; 2.93 (95% C.I.: 2.43–3.54)). However, BNT162b2 vaccinees seemed have a higher risk for serious adverse events than the other two vaccines (crude incidence rate ratio (cIRR); BNT162b2 vs. mRNA1273; 1.50 (95% C.I.: 1.05–2.15; BNT162b2 vs. MVC-COV1901; 2.15 (1.15–4.02)). Similarly, BNT162b2 vaccinees seemed have a higher risk for cardiac adverse events than the other two vaccines (crude incidence rate ratio (cIRR); BNT162b2 vs. mRNA1273; 1.73 (95% C.I.: 1.13–2.63; BNT162b2 vs. MVC-COV1901; 2.99 (1.31–6.82)). On the contrary, the mRNA1273 and BNT162b2 vaccinees were three times more likely to have NSAE than those of MVC-COV1901 (crude incidence rate ratio (cIRR); mRNA1273 vs. MVC-COV1901; 3.81 (95% CI: 3.19–4.55); BNT162b2 vs. MVC-COV1901; 3.06 (95% CI: 2.53–3.72)) (Table 3).

### 3.3. Estimated Incidence Rates (eIR) of Self-Reported Adverse Events (AE) after Booster Dose among Respondents

**Figure 3:**
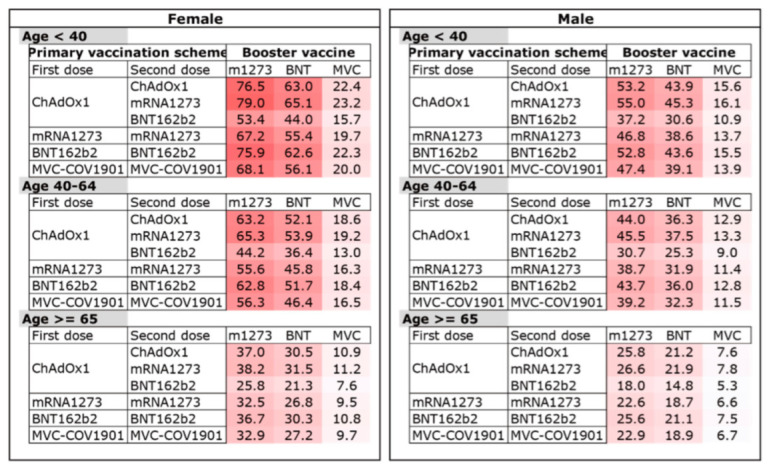
Estimated incidence rates (eIR) of adverse events (AE) among age, sex, primary vaccine and brand of booster vaccine strata. Incidence rates (IR) were estimated using a Poisson regression model and presented as a fraction of 100 respondents. The shading of each cell denotes the IR of each stratum. While a dark color (e.g., dark red) refers to a higher IR, suggesting a higher risk for AE, a light color (e.g., white) refers to a lower rate.

To optimize booster vaccine decision making, the estimated IR (eIR) of different groups was calculated to support booster vaccine decision making (Figure 3). Using the fitted Poisson regression model, we calculated the eIR for every group of combinations of risk factors including gender, age, primary vaccination scheme and booster vaccine. 

In Figure 3, the set of primary vaccine and booster dose, and the eIR of all AE are listed. The darker color in the background denoted the higher eIR of the AE. Because of Taiwanese vaccination policy preference, most respondents received ChAdOx1 as the first dose, but would change to a different brand of vaccine as the second dose. If the respondents received mRNA-1273, BNT162b2 or MVC-COV1901 as the first dose, most of them received the same brand of vaccine as the second dose. Consequently, the response in some primary vaccine combinations was too low to calculate. As we can see, regardless of their age, gender or primary vaccination, the mRNA1273 vaccine had the most AE, and MVC-COV1901 had the least. In both subgroups of homologous and heterologous COVID-19 booster vaccinations, the eIR of AE was only related to the brand of booster dose, irrelevant to the brand of primary doses.

## 4. Discussion

The incidence of AE in booster doses is critical for the successful promotion of the COVID-19 vaccine booster program. The more information that can be realized, the better it is for people to select the appropriate brand of vaccine [2,14]. According to an Israeli study, the third dose of BNT162b2 was linked to modest short-term local and systemic responses, which were more common in younger vaccinees [7]. Another study on primary series found that post-vaccination adverse outcomes in BNT162b2 and ChAdOx1 differed by gender [17]. In our study, VAERS data from hospitals provided real-world evidence to solve the information deficiency. While more than half of the responses (52.2%) reported the presence of AE after receiving a booster dose, only a few AE were considered SAE (2.4%). The reported AE were significantly higher among female respondents under the age of 64 years of age. The strongest factor linked to AE was the brand of a booster vaccine and the incidence of AE in mRNA1273 vaccinees was higher than in BNT162b2 and the incidence of BNT162b2 was higher than MVC-COV1901. The eIR of every group of combination of age, sex, primary vaccine and the brand of booster vaccine was calculated to support the decision making of the booster vaccine. These findings from real world responses may fill the information gap and help to optimize booster vaccine decision making and promote COVID-19 booster vaccination programs.

Our data clearly showed that the IR of AE in the type of mRNA vaccine (mRNA-1273, BNT162b2) was evidently higher than the protein subunit vaccine (MVC-COV1901). Among mRNA vaccines, the IR of AE in mRNA1273 was similarly higher than in BNT162b2. The results were supported by the previous research [18,19,20]. Since there was still a scarcity of information about the protein subunit vaccine, the current finding provides important reference for the gap. In addition to MVC-COV1901, which was made in Taiwan and has only been tested in clinical trials in Taiwan and Paraguay [10], another brand of subunit protein vaccine, Novavax (Nuvaxovid), has been listed on the EUL by WHO and Emergency Use Authorization (EUA). The current result may support policy makers to decide whether or how to adopt subunit protein vaccines into COVID-19 vaccination programs with the increasing availability of protein subunits vaccines.

The decision for the booster vaccine could be easier with the current findings. As there had already been substantial debate on safety and efficacy for booster vaccines [15,16,17,18,19], the decision for the booster vaccine became more complicated since the mix-and-match method policy had introduced more combinations of primary vaccination schemes in the real world, not present in the previous literature [18]. Our result clearly indicated that the issue of the difference in AE between homologous and heterologous COVID-19 booster vaccination should be minimal. Moreover, the table of estimated incidence rate of AE predicted the average incidence rates for groups with a combination of genders, age groups, primary vaccine scheme and brands of booster vaccine. Such information should support making optimal decisions on booster vaccines.

However, the safety of the booster dose in special populations, such as people with autoimmune disease, should be put into particular consideration. One study showed that immune-mediated disease flares or onsets temporally-associated with SARS-CoV-2 vaccination appear rare [21]. Another study revealed that vaccines against SARS-CoV-2 showed good short-term safety in myasthenia gravis patients [22]. The other research showed COVID-19 vaccination is associated with no increased risk of side effects in rheumatic diseases [23]. To date, many studies have revealed the benefits of COVID-19 vaccination in specific diseases. We warranted the decision booster vaccine of people with special disease should consult to experts before using the average incidence rates in the current study.

Our study adopted an online-based questionnaire and self-reported response. There are some limitations to the study. First, the questionnaire may introduce a nonresponse bias because not every recipient prefers or understands how to fill it in through QR codes. In particular, people over the age of 80 found it difficult to operate cellular phones. Furthermore, people are prone to forgetting to respond. As a result, our response rate was only 24.0%, which may cast doubt on the result. Second, recall bias also existed due to memory loss after 7 days. Third, representative bias was presented; these results was only collected from a hospital-based VAERS in Taiwan. Our results may not be able to extrapolate to the general population. Fourth, most foreigners who cannot read traditional Chinese were excluded because of the traditional Chinese questionnaire. Fifth, people under the age of 20 were excluded from the study because they were not eligible for the booster dose at the time of the study. There are only a few studies on young people; further study is needed to provide more data [24,25,26]. Sixth, our result only included short-term AE in the whole population. No long-term AE were analyzed, and did not focus on the special population, including patients with autoimmune disease. The study required more time and further survey details. Finally, despite the restrictions mentioned above, we can find that the results are similar to those of the previous studies about post-vaccination AE in the general population over 20 years old [14]. The reported population’s IR is approximately similar to the Taiwanese population’s rate [8]. We believe that our research can still be a significant reference.

## 5. Conclusions

Although AE were not uncommon for booster vaccines, almost all AE were not serious and predictable. The brand of vaccine is the strongest factor associated with AE in addition to gender and age. The eIR for every group of risk factor combination would help in optimizing booster vaccine decision making and promote booster vaccination programs.

## Figures and Tables

**Table 1:** Definitions and examples of primary-booster vaccination combinations with mix-and-match method.

Primary Vaccine		Booster Dose	Type of Primary-Booster Combination
1st Dose	2nd Dose	3rd Dose	
mRNA1273	mRNA1273	mRNA1273	Homologous booster
mRNA1273	ChAdOx1	ChAdOx1	Homologous booster
mRNA1273	ChAdOx1	mRNA1273	Heterologous booster
ChAdOx1	ChAdOx1	mRNA1273	Heterologous booster
ChAdOx1	mRNA1273	MVC-COV1901	Heterologous booster

**Table 2:** Characteristics and adverse events (AE) reported to hospital-based Vaccine Adverse Event Reporting System (VAERS) for persons who respond a COVID-19 vaccine booster dose from 13 December 2021 to 13 March 2022, at the vaccination station of the Taipei Veterans General Hospital, Taipei, Taiwan (n = 7382, Taipei, Taiwan).

	No. of Respondents		Occurrence of Any Adverse Event		
Factors	Count	(%)	Count	Incidence Rate per 100 Respondents (95% C.I.)	*p*-Value ^2^
Overall	7382	(100.0)	3852	52.2 (51.0–53.3)	
Gender					<0.001
Female	4921	(66.7)	2908	59.0 (57.6–60.3)	
Male	2461	(33.3)	944	38.2 (36.3–40.2)	
Age group					<0.001
<40	3011	(40.8)	1863	61.9 (60.1–63.6)	
<65	3751	(50.8)	1826	48.7 (47.1–50.3)	
≥65	620	(8.4)	163	26.3 (23.0–29.9)	
Primary vaccination scheme					<0.001
ChAdOx1/ChAdOx1	4407	(59.7)	2403	54.5 (53.1–56.0)	
ChAdOx1/mRNA1273	515	(7.0)	332	64.5 (60.2–68.5)	
ChAdOx1/BNT162b2	48	(0.7)	19	39.6 (26.9–53.9)	
mRNA1273/mRNA1273	1438	(19.5)	671	46.7 (44.1–49.2)	
BNT162b2/BNT162b2	550	(7.5)	313	56.9 (52.7–61.0)	
MVC-COV1901/MVC-COV1901	424	(5.7)	114	26.9 (22.9–31.3)	
Type of primary-booster combination ^1^					<0.001
Homologous booster vaccination	2138	(29.0)	1016	47.5 (45.4–49.6)	
Heterologous booster vaccination	5244	(71.0)	2836	54.1 (52.7–55.4)	
Type and brand of booster vaccine					<0.001
RNA-based	6556	(88.8)	3718	56.7 (55.5–57.9)	
mRNA1273	5374	(72.8)	3156	58.7 (57.4–60.0)	
BNT162b2	1182	(16.0)	562	47.5 (44.7–50.4)	
Protein subunit					
MVC-COV1901	826	(11.2)	134	16.2 (13.9–18.9)	

^1^ Type of primary-booster vaccination was determined by serial use of homologous boosters (same vaccine product as the last primary vaccine) and heterologous boosters (different vaccine product from the last primary vaccine) in fully vaccinated recipients. ^2^ Chi-square tests to assess the statistical significance of difference.

**Table 3:** Reports of adverse events (AE) to the hospital-based Vaccine Adverse Event Reporting System (VAERS) by COVID-19 booster vaccine brand among people who received a booster vaccine dose from 13 December 2022 to 13 March 2022, at the Taipei Veterans General Hospital vaccination station, Taipei, Taiwan (n = 7382, Taipei, Taiwan).

	Overall (n = 7382)		mRNA1273 (n = 5374)		BNT162b2 (n = 1182)		MVC-COV1901 (n = 826)		
	Count ^1^	IR ^2^ (%)	Count ^1^	IR ^2^ (%)	Count ^1^	IR ^2^ (%)	Count ^1^	IR ^2^ (%)	*p*-Value ^3^
Total no. of any adverse events	3852	52.2	3156	58.7	562	47.5	134	16.2	<0.001
Serious adverse events (SAE)	174	2.4	121	2.3	40	3.4	13	1.6	0.019
Cardiac symptoms	116	1.6	79	1.5	30	2.5	7	0.8	0.006
Chest pain	81	1.1	52	1.0	26	2.2	3	0.4	<0.001
Short of breath	49	0.7	38	0.7	7	0.6	4	0.5	0.72
Systematic allergic reactions	64	0.9	45	0.8	12	1.0	7	0.8	0.83
Non-serious adverse events (NSAE)	3831	51.9	3147	58.6	557	47.1	127	15.4	<0.001
Local reactions	3483	47.2	2916	54.3	486	41.1	81	9.8	<0.001
Flu like symptoms									
Tiredness	2393	32.4	2018	37.6	323	27.3	52	6.3	<0.001
Headache	1482	20.1	1245	23.2	208	17.6	29	3.5	<0.001
Fever	1319	17.9	1163	21.6	147	12.4	9	1.1	<0.001
Chillness	139	1.9	117	2.2	21	1.8	1	0.1	<0.001
Cardiac symptoms									
Palpitation	78	1.1	46	0.9	24	2.0	8	1.0	0.002
Gastrointestinal symptoms									
Nausea	66	0.9	56	1.0	8	0.7	2	0.2	0.052
Muscle/joint pain	374	5.1	307	5.7	56	4.7	11	1.3	<0.001
Menstrual problems	12	0.2	9	0.2	1	0.1	2	0.2	0.68
Others	288	3.9	193	3.6	63	5.3	32	3.9	0.02

^1^ The total number of the reported events exceeded the total of respondents reporting any adverse event (AE) because one booster dose recipient may report over one AE. ^2^ Incidence rates (IR) were calculated as the sum of all reported adverse events divided by the number of respondents and expressed as a fraction of 100 respondents. ^3^ A Poisson regression model was used that uses each type of AE occurrence as the dependent variable and the booster vaccine brand as the independent variable to test the statistical significance of incidence rate of AE among three brands of COVID-19 vaccine boosters.

## Appendix A

**Table A1:** The component and adjuvants of four brands of COVID-19 vaccines for booster dose *.

Vaccine	Available Formulation	Component	Recommend Age
ChAdOx1	5 mL multidose vial ^1^	Each dose (0.5 mL): Chimpanzee Adenovirus encoding the SARS-CoV-2 Spike glycoprotein (ChAdOx1-S), not less than 2.5 × 10^8^ infectious units (Inf.U). List of excipients: L-Histidine; L-Histidine hydrochloride monohydrate; Magnesium chloride hexahydrate; Polysorbate 80 (E 433); Ethanol; Sucrose; Sodium chloride; Disodium edetate (dihydrate); Water for injections.	≥18 years
mRNA1273	5 mL multidose vial ^1^	Each dose (0.25 mL): 50 μg of elasomeran, a COVID-19 mRNA Vaccine (embedded in SM-102 lipid nanoparticles). List of excipients: SM-102(heptadecan-9-yl 8-{(2-hydroxyethyl)[6-oxo-6-(undecyloxy)hexyl]amino}octanoate); Cholesterol; 1,2-distearoyl-sn-glycero-3-phosphocholine (DSPC); 1,2-Dimyristoyl-rac-glycero-3-methoxypolyethylene glycol-2000 (PEG2000 DMG); Trometamol; Trometamol hydrochloride; Acetic acid; Sodium acetate trihydrate; Sucrose; Water for injections.	≥18 years
BNT162b2	0.45 mL multidose vial ^2^ diluted in its original vial with 1.8 mL sodium chloride 9 mg/mL (0.9%) solution for injection	Each dose (0.3 mL): 30 μg of tozinameran, a BNT162b2 RNA (embedded in lipid nanoparticles). List of excipients: ALC-0315 = (4-hydroxybutyl) azanediyl)bis (hexane-6,1-diyl)bis(2-hexyldecanoate); ALC-0159 = 2-[(polyethylene glycol)-2000]-N,N-ditetradecylacetamide; 1,2-Distearoyl-sn-glycero-3-phosphocholine; Cholesterol; Potassium chloride; Potassium dihydrogen phosphate; Sodium chloride; Disodium hydrogen phosphate dihydrate; Sucrose; Water for injections.	≥12 years
MVC-COV1901	0.5 mL syringe. 5 mL multidose vial ^1^	Each dose (0.5 mL): 15 μg of SARS-CoV 2 recombinant spike protein. List of excipients: CpG 1018; Aluminum hydroxide; Phosphate buffer solution.	≥20 years

^1^ Multidose vials contain 10 doses. ^2^ Multidose vials contain 6 doses. * The information was acquired from the webpage of Taiwan Food and Drug Administration [27].
